# 聚焦肿瘤治疗，关注其他疾病

**DOI:** 10.3779/j.issn.1009-3419.2018.04.01

**Published:** 2018-04-20

**Authors:** 伦旭 刘, 克能 陈, 树庚 高

继续教育，专题讲座一直以来都是我们协会年会担负的重要看点。

1、临床数据标准化。上期张逊会长畅谈了大数据互联网时代胸外科的美好未来与机遇，本期我们约请了身兼专家和院长双重角色的姜杰教授的专题《基于标准化术语的胸外科结构化电子病例实践》，是胸外科迎接大数据时代的具体体现，也是互联网时代的必由之路。相信会对大家有所帮助。

2、继续教育多样化。信息发展一日千里，科学进步从未停止，毕业后的继续教育始终伴随我们的成长。李小飞教授所在的大学是我国著名的医学高等教育中心，相信他的专题《微课在胸腔外科临床教学中的应用》为年轻医师的培养提供了多样化的选择。

3、热点技术模块化。单孔腔镜手术是近来大家关注的热点技术，本期由知名单孔腔镜肺切除专家朱余明教授倾注的专题《单孔胸腔镜技术培训的思考》，供年轻学者在成长中借鉴与欣赏。

4、罕见病研究多中心化。相对于肺癌食管癌的发病率，胸腺肿瘤是少见疾病，临床证据少，研究困难。ChART组织的多中心回顾研究研究数据，已在国际胸腺肿瘤治疗指南的制定中发挥重要影响。敬请关注ChART发起人，CSCO纵隔肿瘤专业委员会主任委员方文涛教授有关胸腺微创手术的专题《胸腺肿瘤微创手术研究进展》。

5、特殊技术需要传承。早期发现，早期治疗，一直是大家的理想，但仍然有许多患者在诊断时就已是局部晚期甚或已有全身转移，对他们的治疗需要高超的外科技术，更需要良好的肿瘤全身治疗理念。许林教授的特约专题《恶性胸腺瘤侵犯上腔静脉的外科治疗》和钟文昭教授的专题《随访观察——主病灶切除的胸腔内播散腺癌或鳞癌患者的可选治疗策略》就是这类典范，希望对大家能有帮助。

6、现代胸科跨界化。多学科/跨学科综合学问、技术和人才是当今的主旋律。李小飞教授、肖高明教授都是胸壁缺损修复的知名专家。他们的专题《胸壁骨性重建的研究进展》涉及新型材料学、整形外科学等一系列跨界的理论、技术与知识，相信一定会对大家有所帮助。

7、聚焦肿瘤治疗，关注其他疾病。肿瘤已然是胸外科的主要疾病，但是胸外科的范畴远不止癌症，胸外科临床也需要其他知识。为此，本期约请了与我们胸外科日常工作紧密相关的专题——结核病的治疗。京沪两地结核病专家专题评述了肺结核的外科治疗，为大家的临床提供帮助；HIV阳性合并肺癌的治疗也已摆在我们面前，值得关注；外科感染的处理是外科人永久的话题，希望本期的相关内容惠及有大家的临床实践。

总之，正如赫捷院士在寄语中指出的那样，协会是我们大家的"家" ，是聚人、聚气、聚事的平台，欢迎批评，欢迎赐教，期盼未来！

